# Screening and Confirmatory Testing for SARS-CoV-2 Antibodies: Comparison of Health and Non-Health Workers in a Nationwide Healthcare Organization in Central Europe

**DOI:** 10.3390/jcm10091909

**Published:** 2021-04-28

**Authors:** Johann Bartko, Sonja Zehetmayer, Lukas Weseslindtner, Karin Stiasny, Andrea Schloegl, Ernst Forjan, Elisabeth Zwettler, Andreas Krauter, Felix Keil, Nazanin Sédille-Mostafaie

**Affiliations:** 1 Institute for Laboratory Medicine, Hanusch Hospital, 1140 Vienna, Austria; andrea.schloegl@oegk.at (A.S.); nazanin.sedille-mostafaie@oegk.at (N.S.-M.); 2 Center for Medical Statistics, Informatics, and Intelligent Systems, Medical University of Vienna, 1140 Vienna, Austria; sonja.zehetmayer@meduniwien.ac.at; 3 Center for Virology, Medical University of Vienna, 1090 Vienna, Austria; lukas.weseslindtner@meduniwien.ac.at (L.W.); karin.stiasny@meduniwien.ac.at (K.S.); 4 Department of Haemato-Oncology, Hanusch Hospital, 1090 Vienna, Austria; ernst.forjan@oegk.at (E.F.); felix.keil@oegk.at (F.K.); 5 Medical Directorate, Hanusch Hospital, 1140 Vienna, Austria; elisabeth.zwettler@oegk.at; 6 Medical Services, Austrian Health Insurance Fund, 1030 Vienna, Austria; andreas.krauter@oegk.at

**Keywords:** SARS-CoV-2, COVID-19, health personnel

## Abstract

Despite being located close to the European epicenter of the COVID-19 pandemic in Italy, Austria has managed to control the first wave. In Austria, the largest health insurance fund covers 7 million people and has 12,000 employees, including 3700 healthcare workers (HCW). For patient and staff safety, transmission control measures were implemented and mass testing of employees for SARS-CoV-2 antibodies was conducted. An IgG SARS-CoV-2 rapid test on fingerstick blood was used as a screening test (ST), followed by serologic studies with 3 different immunoassays and confirmatory testing by a neutralization test (NT). Among 7858 employees, 144 had a positive ST and 88 were confirmed by a NT (1.12%, CI: 0.9–1.38%). The positive predictive value (PPV) of the ST was 69.3% (CI: 60.5–77.2). Interestingly, 40% of the NT positive serum samples were tested negative in all 3 immunoassays. Of the total sample, 2242 HCW (28.5%) were identified. Unexpectedly, there was no difference in the prevalence of NT positives in HCW compared to non-HCW (23/2242 vs. 65/5301, *p* = 0.53). SARS-CoV-2 antibody prevalence was not increased among HCW. Although HCW are at potentially increased risk for SARS-CoV-2 infection, transmission control measures in healthcare facilities appear sufficient to limit transmission of infection.

## 1. Introduction

By the end of February 2020, the first reports about the serious situation at intensive care units in northern Italy emerged through social media. Austria, sharing a direct border to Italy, was among the first countries in Europe to initiate a nationwide lockdown. With approximately 8.9 million inhabitants, Austria′s healthcare system provides care to a population size comparable with a state in the US. The largest health insurance fund in Austria covers more than 7 million people and has approximately 12,000 employees. Within our organization, nearly 3700 healthcare workers (HCW) care for over 500,000 patients per year. During this first phase of the pandemic, healthcare services and processes were adapted to ensure patient and staff safety. Measures such as personal protective equipment (PPE), visitor restriction, isolation, and transfer of COVID-19 patients to dedicated COVID-19 hospitals, telemedicine-based outpatient services, and SARS-CoV-2 screening of non-COVID-19 patients prior to admission were implemented. Interestingly, the volume of patients dropped significantly from the very beginning of the pandemic, an observation that has also been made by other investigators [1,2]. Despite extensive infection control efforts, safety concerns may have led to patient’s change of care seeking behavior. Indeed, high COVID-19 infection rates have been reported among HCW from China and Italy [3,4]. Given the large numbers of asymptomatic or mild cases of SARS-CoV-2 infections [5], many of our HCW may have had undetected infections. Therefore, we assessed the prevalence of SARS-CoV-2 IgG antibodies among employees of the Austrian Health Insurance fund and compared HCW with non-HCW. These data provide the basis for resource management, strategic planning, and successful implementation of a COVID-19 vaccination program.

## 2. Materials and Methods

### 2.1. Participants

Employees of the Austrian Health Insurance fund were recruited by mass mailings and announcements in the institution′s intranet. Subjects who were at least 18 years of age were eligible for inclusion in the study. Healthcare workers included physicians, nurses, nursing assistants, phlebotomists, radiology technicians, dietitian, dental health professionals, and physical/occupational therapists at a 400-bed tertiary referral hospital, five large multi-specialty outpatient clinics, and nearly a hundred primary care services across Austria. Until the end of the study, the tertiary referral hospital had no dedicated COVID-19 unit with airborne infection isolation and was not designated for the treatment of patients with COVID-19. Non-healthcare workers included employees not directly exposed to potentially infectious patients, such as personnel of dietary/environmental/IT services, maintenance, laundry, and engineering facilities or personnel working in office buildings.

### 2.2. Study Design

**Figure 1:**
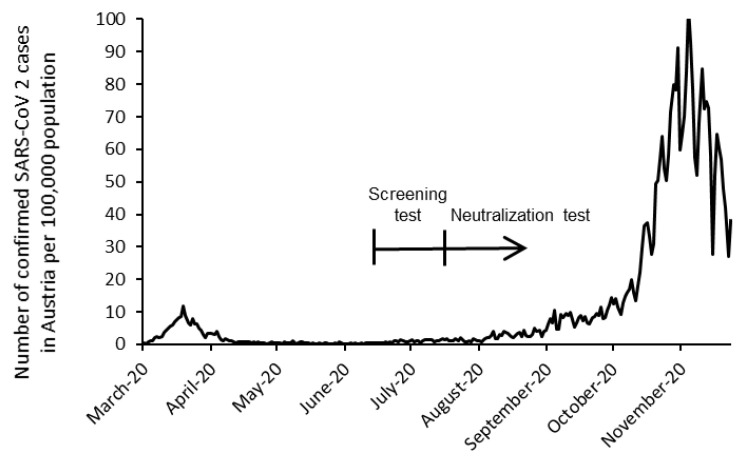
Timeline of the study in context of the epidemiologic situation in Austria.

In this prospective cross-sectional study, SARS-CoV-2 antibody prevalence was assessed among employees of the Austrian Health Insurance fund. The study was reviewed and approved by the ethics committee of the City of Vienna (EK-20-154-VK) and was conducted in accordance with the ethical standards laid down in the Declaration of Helsinki. During July 2020, participants were tested for SARS-CoV-2 antibodies by analysis of fingerstick whole blood. Participants were asked about their occupation, potential symptoms of COVID-19 in the last months, previous testing for SARS-CoV-2 infection by PCR test (swab from nose/throat), and comorbidities associated with an increased risk of severe COVID-19 by a paper-based questionnaire. In participants with a positive screening test, a second blood draw was carried out within 4 weeks for determination of serum-neutralizing activity (Figure 1).

### 2.3. Outcomes

The primary outcome was SARS-CoV-2 IgG antibody prevalence among employees of the Austrian Health Insurance fund. In participants with a confirmed, reactive screening test result, we compared baseline characteristics, antibody prevalence and potential COVID-19 symptoms of HCW with non-HCW.

### 2.4. Screening and Confirmatory Testing

For screening, a commercially available lateral flow test (LFT) detecting IgG antibodies against the SARS-CoV-2 nucleocapsid (N) protein was used as a screening test (ST). The ST was performed by trained clinical laboratory and nursing staff members in accordance with the manufacturers’ instructions for fingerstick whole blood (Panbio COVID-19 IgG/IgM Rapid Test Device, Abbott Rapid Diagnostics, Jena, Germany). In contrast to IgG class antibodies, IgM antibody testing appears less useful for detection of past infection, given the poor sensitivity [6] and potential cross-reactivity with other coronaviruses. Thus, only reactive IgG test results were considered positive in the study. For safety reasons, subjects with a solitary and strong IgM test line intensity underwent further nucleic acid amplification testing (NAAT) for SARS-CoV-2 RNA from nasopharyngeal swabs. However, there were no positive SARS-CoV-2 PCR tests among subjects with a reactive IgM test result. Reported sensitivities and specificities of the ST range from 60.7 to 98.7% and from 98.1 to 100%, respectively, in the literature [6,7,8,9]. However, regarding the use of finger prick testing, there are very limited data available. A seroprevalence survey showed a sensitivity of 77% in 43 patients [10].

For confirmation of the ST, serum samples were drawn within 4 weeks from participants who had a reactive IgG LFT result and a neutralization test (NT) was performed by an in-house neutralization assay measuring the antibody-mediated inhibition of cytopathic effects (CPE) in SARS-CoV-2 infected Vero-E6 cells, as previously described [11,12]. Briefly, serial dilutions of heat-inactivated serum samples were incubated with 50–100 TCID50 SARS-CoV-2 for 1 h at 37 °C before the mixture was added to Vero E6 cells (ATCC^®^ CRL-1586, Manassas, VA, USA), followed by incubation for 2–3 days. NT titers were expressed as the reciprocal of the serum dilution that protected against virus-induced cytopathic effects. NT titers ≥ 10 were considered positive. In addition, serologic studies with 3 different immunoassays were performed according to the manufacturers’ instructions (LIAISON^®^ SARS-CoV-2 S1/S2 (DiaSorin S.p.A., Saluggia, Italy) on the LIAISON^®^ XL Analyzer; Anti-SARS-CoV-2-ELISA IgG (EUROIMMUN Medizinische Labordiagnostika AG, Lübeck, Germany) on the EUROIMMUN Analyzer I; SARS-CoV-2 IgG (Abbott Laboratories, Chicago, IL, USA) on the ARCHITECT i System).

### 2.5. Statistics

Absolute and percentage frequencies and 95% Clopper–Pearson confidence intervals (CI) were calculated for binary variables and the positive predictive value and corresponding 95% confidence intervals were calculated for the ST (gold standard NT). Cross tables were given for the NT test versus the other tests. A Chi-square test was applied to test the difference in proportion of HCW between ST positive and NT positive and negative participants. The significance level was set to 0.05. The prevalence and 95% CI in our sample was compared with the prevalence of the Austrian population at the 31 July and the 31 August (data from https://www.data.gv.at/katalog/dataset/covid-19-zeitliche-darstellung-von-daten-zu-covid19-fallen-je-bundesland/resource/7669768d-b035-4d66-ad2b-3771b7639588, 21 January 2021). All analyses were performed with R version 3.6.3 (R Foundation for Statistical Computing, FreeSoftware Foundation, Boston, MA, USA).

## 3. Results

**Figure 2:**
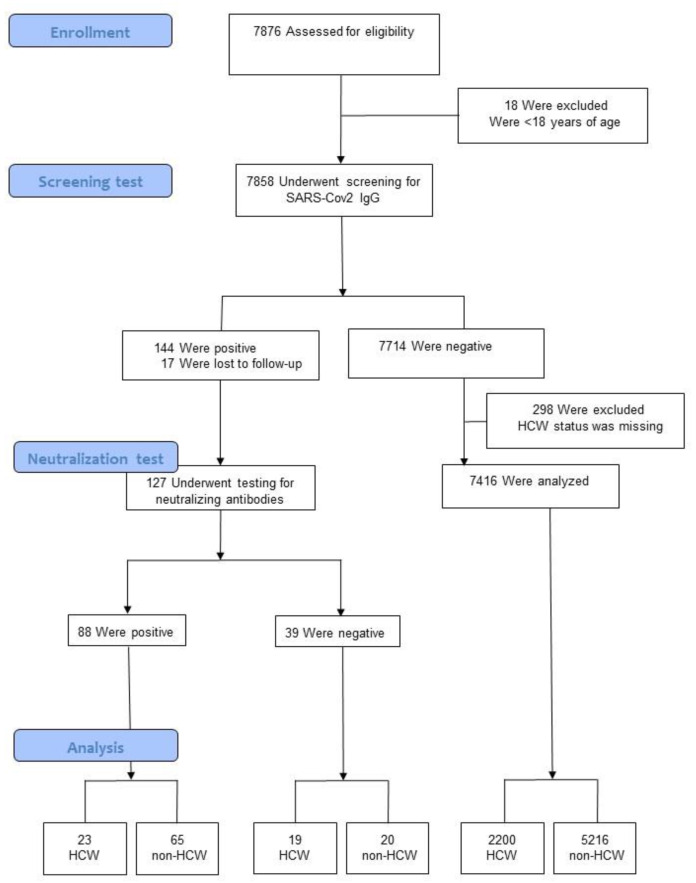
Flow diagram. Healthcare workers: HCW.

All employees were invited to participate in the study (12,157 persons); of those, 7876 (65%) gave written informed consent to participate and 18 subjects, who were younger than 18 years of age (apprentices), were excluded. A flow diagram of the study population is summarized in Figure 2. Participants had a mean age of 43.9 years (SD = 11) and 5626 of them (71.6%) were female.

### 3.1. Screening Test (ST)

Of the 7858 eligible subjects who were tested, 144 (1.83%, CI: 1.55–2.15) had a positive ST, i.e., a reactive SARS-CoV-2 IgG antibody LFT result. They had a mean age of 45.2 years, and 103 of them (71.5%) were female. Of the 7714 employees who had a negative ST, the mean age was 43.9 years and 5523 of them (71.6%) were female.

### 3.2. Confirmatory Testing (Neutralization Test, NT)

From the 144 participants who tested positive with the ST, 17 subjects did not undergo serologic confirmatory testing (due to personal reasons), whereas 127 were further tested for neutralizing antibodies against SARS-CoV-2. Neutralizing antibodies were detectable in 88 participants, which corresponds to 1.12% (CI: 0.9–1.38%) of the total sample (88/7841). The mean age was 45.5 years and 57 were female (64.8%, CI: 53.9–74.7 versus 71.6%, CI: 70.6–72.6 in the ST negative group with a mean age of 43.9 years).

### 3.3. Healthcare Workers

Of the total sample (7858), 298 subjects with missing HCW status and 17 subjects who did not undergo serologic confirmatory testing were excluded from the final analysis (Figure 2). The seroprevalence (positive NT) among HCW was 1.0% (CI: 0.7–1.5), or 23 of 2242 subjects. The prevalence among non-HCW was 1.2% (CI: 0.9–1.6), or 65 of 5301 subjects. Basic characteristics of HCW and non-HCW are provided in Table 1.

There was no difference in the seroprevalence between the HCW group and the non-HCW group (*p* = 0.53, Chi-square test). Because of the traditional paper-based questionnaire, only detailed characteristics of HCW and non-HCW who had detectable neutralizing antibodies were analyzed and are provided in Table 2.

### 3.4. Symptoms

**Figure 3:**
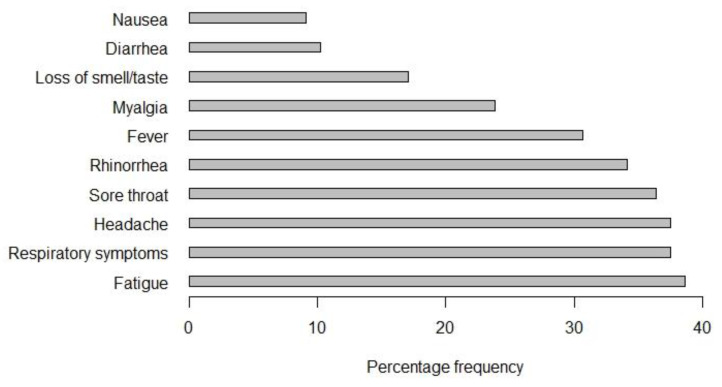
Percentage frequencies of common symptom reported by individuals with SARS-CoV-2 neutralizing antibodies.

Of the 88 participants who had detectable neutralizing antibodies, 29 participants (33%, CI: 23.3–43.8) reported no symptoms. Frequencies of common symptoms are provided in Figure 3.

Among HCW who tested positive with the NT, 5 of 23 (22%) were asymptomatic compared to 24 of 65 (37%) in the non-HCW group.

### 3.5. PCR

Of the 88 participants who had detectable neutralizing antibodies, 23 had been previously tested for SARS-CoV-2 RNA and 13 of them had a positive result. The median time from previous PCR testing to the ST was 113 days (range 99 to 131) for positive PCR test results and 82 days (range 16 to 132) for negative PCR test results. All subjects with a positive SARS-CoV-2 RNA test result reported symptoms compatible with COVID-19. Among the 39 individuals who were tested negative with the NT, 5 subjects had been tested previously for SARS-CoV-2 RNA and all of them had negative PCR test results.

### 3.6. Performance of the Screening Test

A total of 88 of the ST positive individuals were also positive according to the NT (69.3%, CI: 60.5–77.2). Under the assumption, that NT is the gold standard of being positive, this percentage reflects the positive predictive value of the ST.

### 3.7. Serologic Immunoassays

Of the 39 serum samples tested negative with the NT, 97, 100, and 95% were negative with DiaSorin, Euroimmun, and Abbott (Table 3). Of the 88 samples tested positive with the NT, only 59, 58, and 57% were positive for DiaSorin, Euroimmun, and Abbott. There were 35 serum samples (40%) which tested negative in all three immunoassays despite being positive with the NT.

## 4. Discussion

In early March, an alpine ski resort, linked to a number of COVID-19 cases across Europe after skiers returned home, became the center of a COVID-19 outbreak in Austria [13]. A nationwide lockdown was imposed, and Austria’s healthcare system prepared for the surge. Healthcare facilities of the Austrian Health Insurance fund had to adapt structures and processes to respond to the changing environment. As part of the safety concept, testing for SARS-CoV-2 IgG antibodies was offered to all employees to determine the seroprevalence among employees at higher risk for exposure to SARS-CoV-2 and employees with low exposure risk.

In our study, the overall positivity rate of confirmed cases was 1.12%, which is higher compared to the prevalence in the Austrian population at similar time points (0.24% by 31 July and 0.31% by 31 August). Although we observed higher percentages than in the Austrian population, some limitations may have a bias on the comparison. Data from the Austrian population is based on PCR-testing results and includes more individuals with symptoms. Thus, the difference may be due to a higher detection rate of asymptomatic/unreported cases in our population and possible false-negative molecular testing of swab specimen [14,15]. However, a higher seroprevalence was also reported by other investigators who detected virus-specific antibodies in 1.88% of a representative collective of working adults after the first lockdown in Austria [16]. Clinically, one-third of employees who had detectable neutralizing antibodies reported no symptoms compatible with COVID-19, which is comparable to the proportion of asymptomatic infections found by others [17]. Frequencies of common and less common symptoms were also similar to the findings in other studies [18].

Another important question of our study was whether the prevalence of SARS-CoV-2 antibodies was higher among HCW, which may be caused by higher risk of transmission of SARS-CoV-2 [19,20,21,22] or SARS-CoV-2 RNA contamination of surfaces of hospital environments [23] or air samples from both ICU and general wards [24]. Unexpectedly, we found no difference in the prevalence between HCW and non-HCW. These findings contrast with previous studies showing increased seroprevalences of SARS-CoV-2 during the initial phase of the pandemic among HCW in China, UK, and Sweden [25,26,27]. Several factors may have influenced our results. First, a nationwide lockdown was adopted early in Austria and the overall infection rates stayed relatively low (<10 reported cases per 100,000 inhabitants). Secondly, transmission control measures such as wearing PPE were rapidly implemented in healthcare facilities across the country. Thirdly, our population did not include HCW from designated COVID-19 hospitals as isolation and transfer of COVID-19 patients to dedicated COVID-19 hospitals was a key component of the patient safety strategy. However, recently published data from the US and Germany are more consistent with our findings. Two studies from Germany detected SARS-CoV-2 IgG antibodies in only 1.6 and 1.8% of HCW, including HCW with a high exposure risk [28,29]. A seroprevalence study in the NYC area investigated a large cohort of 46,117 HCW [30]. Although the prevalence was 10 times higher (13.7%) than in our sample, it was similar to the statewide prevalence [31]. These data indicate that even in a higher prevalence/high exposure setting, transmission control measures are effective in preventing the spread of COVID-19 in healthcare centers. Yet, this may be different in other geographic locations or at a more advanced period of the pandemic. With the persistence of the COVID-19 pandemic, rates of healthcare associated SARS-CoV-2 infections are expected to increase. Thus, a longer period of observation may show different results. In most seroprevalence studies, an appropriate control group is absent [32]. An important feature of our study design was the inclusion of a control group of working adults, which allowed for comparison of HCW with non-HCW. Further strengths of the study were its use of a large and nationally representative sample and the use of virus microneutralization assays for confirmatory testing. For large-scale screening, rapid tests are needed [33]. In terms of the feasibility, our study shows that using a rapid test device on fingerstick whole blood is a practical method for mass testing. Our study comes not without limitations; particularly, PCR data were only available in ~25% of serologically confirmed cases, indicating a relative large number of undetected cases, as reported by other investigators [34]. Further limitations include a time lag between ST and NT (no longer than four weeks) and that the sensitivity or specificity of the ST could not be calculated as the NT was only performed in individuals with positive ST. However, as the NT is considered the gold standard of being positive [35], 69.3% of the ST positive people were also positive according to the NT. This percentage also reflects the PPV of the ST in our cohort. The PPV is also dependent on the pretest probability (prevalence). Thus, the PPV will rise when the prevalence increases [36]. Although the PPV of the ST will increase with rising infection rates, a single screening test approach in low-prevalence situations may be appropriate for SARS-CoV-2 serosurveillance studies, but is limited in the evaluation of an individual’s protective immunity against SARS-CoV-2. Interestingly, 40% of the NT positive serum samples were tested negative in all three immunoassays. Serologic assays sensitivities usually range from 80 to over 90% in larger cohorts [37,38]. Although IgG antibodies remain detectable for months after infection [39], this may be different in the mild/asymptomatic COVID-19 population as most data are obtained from hospitalized patients [40,41]. Therefore, commercial serology tests may have limited clinical value particular in individuals with low antibody response.

## 5. Conclusions

Overall, we could demonstrate that the SARS-CoV-2 antibody prevalence was not increased among HCW compared to non-HCW. Our data clearly show that healthcare services were safely provided to patients during this period of the COVID-19 pandemic. The study may also provide important information for management of personnel resources and vaccination strategies.

## Figures and Tables

**Table 1:** Characteristics of healthcare workers (HCW) and non-healthcare workers who underwent screening for SARS-CoV-2 antibodies and completed the study.

	HCW (*n* = 2242)	Non-HCW (*n* = 5301)
Age Group, *n* (%)		
18–39 years	1071 (48)	1399 (26)
40–49 years	589 (26)	1502 (28)
50–59 years	521 (23)	2143 (41)
60–69 years	61 (3)	257 (5)
Gender, *n* (%)		
Female	1118 (50)	4194 (79)
Male	1124 (50)	1107 (21)

**Table 2:** Characteristics of healthcare workers and non-healthcare workers who had detectable neutralizing antibodies.

	Healthcare Workers (*n* = 23)	Non-Healthcare Workers (*n* = 65)
Age group, *n* (%)		
18–39 years	8 (35)	14 (22)
40–49 years	6 (26)	23 (35)
50–59 years	9 (39)	26 (40)
60–69 years	0	2 (3)
Gender, *n* (%)		
Female	15 (65)	42 (65)
Male	8 (35)	23 (35)
Job function, *n* (%)		
Nurse	9 (39)	–
Nursing assistance	4 (17)	–
Physician	4 (17)	–
Physiotherapist	2 (9)	–
Dental assistance	2 (9)	–
Other healthcare personnel	2 (9)	
Administrative and clerical	–	59 (91)
Cleaning service	–	4 (6)
Dietary service	–	1 (1.5)
Maintenance service	–	1 (1.5)
Chronic medical conditions, *n* (%)		
Hypertension	6 (26)	6 (9)
Cancer	0	0
Diabetes mellitus	0	3 (5)
Chronic lung disease	2 (9)	3 (5)
Autoimmune disease	1 (4)	2 (3)
Cardiac disease	1 (4)	1 (1.5)
Previous PCR testing, *n* (%)		
Positive	5 (22)	8 (12)
Negative	4 (17)	6 (9)
Not performed	14 (61)	51 (79)

**Table 3:** Cross table NT results versus 3 different immunoassays.

Neutralization Test	Level	Negative	Positive
*n*		39	88
Diasorin (%)	neg	38 (97.4)	52 (59.1)
	pos	1 (2.6)	36 (40.9)
Euroimmun (%)	neg	39 (100)	51 (58)
	pos	0 (0)	37 (42)
Abbott (%)	neg	37 (94.9)	50 (56.8)
	pos	2 (5.1)	38 (43.2)

## Data Availability

The data presented are available and can be provided from the corresponding author on request.
